# Cellular Mechanisms Underlying Eosinophilic and Neutrophilic Airway Inflammation in Asthma

**DOI:** 10.1155/2015/879783

**Published:** 2015-03-23

**Authors:** Girolamo Pelaia, Alessandro Vatrella, Maria Teresa Busceti, Luca Gallelli, Cecilia Calabrese, Rosa Terracciano, Rosario Maselli

**Affiliations:** ^1^Department of Medical and Surgical Sciences, University “Magna Græcia” of Catanzaro, Viale Europa, Località Germaneto, 88100 Catanzaro, Italy; ^2^Department of Medicine and Surgery, University of Salerno, Via Salvador Allende, Baronissi, 84081 Salerno, Italy; ^3^Department of Health Science, University “Magna Græcia” of Catanzaro, Viale Europa, Località Germaneto, 88100 Catanzaro, Italy; ^4^Department of Cardiothoracic and Respiratory Sciences, 2nd University of Naples, Via Leonardo Bianchi, 80131 Naples, Italy

## Abstract

Asthma is a phenotypically heterogeneous chronic disease of the airways, characterized by either predominant eosinophilic or neutrophilic, or even mixed eosinophilic/neutrophilic inflammatory patterns. Eosinophilic inflammation can be associated with the whole spectrum of asthma severity, ranging from mild-to-moderate to severe uncontrolled disease, whereas neutrophilic inflammation occurs mostly in more severe asthma. Eosinophilic asthma includes either allergic or nonallergic phenotypes underlying immune responses mediated by T helper (Th)2 cell-derived cytokines, whilst neutrophilic asthma is mostly dependent on Th17 cell-induced mechanisms. These immune-inflammatory profiles develop as a consequence of a functional impairment of T regulatory (Treg) lymphocytes, which promotes the activation of dendritic cells directing the differentiation of distinct Th cell subsets. The recent advances in the knowledge of the cellular and molecular mechanisms underlying asthmatic inflammation are contributing to the identification of novel therapeutic targets, potentially suitable for the implementation of future improvements in antiasthma pharmacologic treatments.

## 1. Introduction

**Figure 1:**
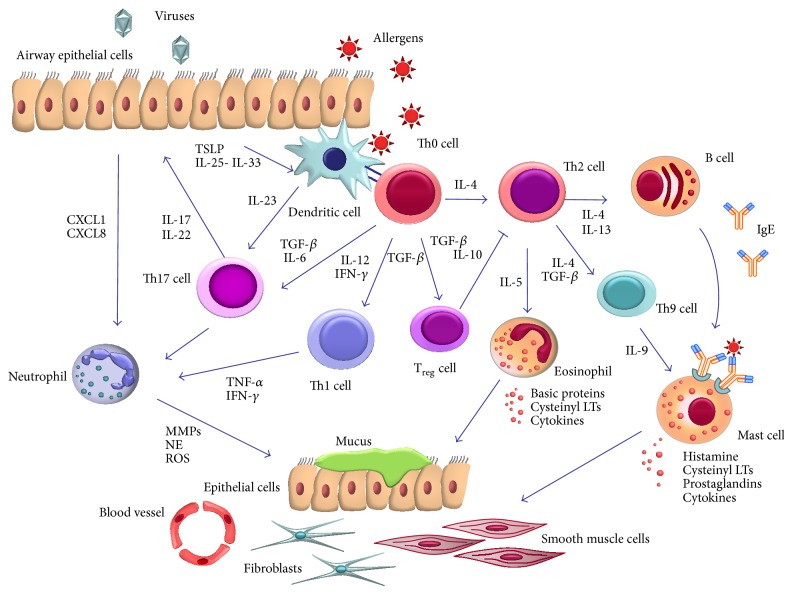
Pathobiology of airway inflammation in asthma. Asthma originates from complex interactions between genetic factors and environmental agents such as aeroallergens and respiratory viruses. In particular, within the airway lumen allergens can be captured by dendritic cells, which process antigenic molecules and present them to naïve (Th0) T helper cells. The consequent activation of allergen-specific Th2 cells is responsible for the production of IL-4 and IL-13 that promote B-cell operated synthesis of IgE antibodies; moreover, Th2 cells also release IL-5, which induces eosinophil maturation and survival. These events are noticeably favoured by a functional defect of IL-10/TGF-*β* producing T regulatory (Treg) cells that normally exert an immunosuppressive action on Th2 cell-mediated responses. In addition to Th2 cells, IL-9 releasing Th9 cells can also undergo activation, thus leading to the growth and recruitment of mast cells, which upon IgE-dependent degranulation release both preformed and newly synthesized mediators. Other important T lymphocytes contributing to asthma pathobiology are Th17 cells, producing IL17A and IL-17F which cause neutrophil recruitment and expansion. Furthermore, IL-12-dependent and IFN-*γ* releasing Th1 cells can be activated, especially as a result of airway infections sustained by respiratory viruses. Finally, many mediators, cytokines, and growth factors produced by several different cells involved in chronic asthmatic inflammation may also affect the functions and proliferation rates of airway structural cellular elements including epithelial cells, fibroblasts, smooth muscle cells, and endothelial vascular cells. See text for further details.

Airway inflammation, together with bronchial hyperresponsiveness and airway structural remodelling, is one of the prominent features of asthma, a phenotypically heterogeneous chronic respiratory disease that affects over 300 million asthmatic people worldwide, who will probably become more than 400 million by 2020 [1–5]. A very large array of cytokines, chemokines, and other proinflammatory mediators, released by both immune-inflammatory and airway structural cells, significantly contribute to shape several different asthma phenotypes [6–8]. Eosinophils are the major inflammatory cells involved in the pathobiology of asthma; indeed, their maturation, activation, survival, and recruitment within the bronchial wall and airway lumen are key pathogenic events implicated in the development of both allergic and nonallergic asthmatic phenotypes [9, 10]. Eosinophilic asthma arises from complex immunologic and proinflammatory mechanisms, mainly orchestrated by T helper (Th)2 lymphocytes, which release interleukins (IL-5, IL-4, and IL-13) (Figure 1). In addition to being driven by adaptive immune mechanisms, Th2-mediated airway eosinophilia can be also associated with relevant innate immune responses, which depend on intercellular communications involving dendritic cells, bronchial epithelial cells, and innate lymphoid cells [11, 12]. Whilst bronchial eosinophilic infiltration is mostly responsible for mild-to-moderate asthma, more severe disease is frequently sustained by mixed patterns of inflammation including both eosinophils and neutrophils, with the latter that can often represent the predominant inflammatory cells detectable in the induced sputum obtained from patients experiencing uncontrolled asthmatic symptoms and exacerbations. Neutrophilic airway inflammation, associated with severe asthma, is triggered by Th1 and especially Th17 lymphocytes (Figure 1) [13, 14]. Within this pathophysiologic context, a key role is played by dendritic cells, which direct the differentiation processes leading to polarization towards the various Th subsets. In particular, commitment to the distinct Th lineages is crucially dependent on the particular airway milieu consisting of specific costimulatory molecules and cytokines, which drive the different Th cell programs [15].

On the basis of the above considerations, the aim of this review article is to provide a synthetic and updated overview of the main pathobiological aspects underlying the development of eosinophilic and neutrophilic airway inflammation in asthma.

## 2. Eosinophilic Asthma

Eosinophilic airway inflammation characterizes the clinical phenotype of early-onset allergic asthma, as well as the occurrence of late-onset nonallergic asthma.

Allergic asthma is triggered by an immune-inflammatory response driven by Th2 cells. This so-called “Th2-high” or “Th2-like” subphenotype of asthma originates from a complex interplay between the innate and adaptive branches of immune system [16, 17]. Differentiation of Th2 lymphocytes requires the cooperation of several promoting factors, including costimulatory molecules and cytokines expressed by dendritic cells and inflammatory cells. In particular, aeroallergens that cause allergic asthma, such as pollens, house dust mite, and animal dander, often have proteolytic properties and also contain trace amounts of bacterial constituents such as lipopolysaccharide (LPS) [18]. Therefore, upon penetration into the airway epithelium, inhaled allergens can activate the Toll-like receptor (TLR) class of pattern recognition receptors involved in innate immunity. TLR activation induces the epithelial synthesis of innate cytokines, such as thymic stromal lymphopoietin (TSLP), IL-25, and IL-33, able to induce the development of Th2 adaptive responses (Figure 1). IL-33 can also be produced by dendritic cells [19]. Furthermore, TLR stimulation also elicits the epithelial release of C-C chemokine ligands 2 (CCL2) and 20 (CCL20), which promote the recruitment and maturation of dendritic cells [16]. The latter extend their intraepithelial processes into the airway lumen, capture aeroallergens, and process them inside the cytoplasm, thus generating allergenic peptide fragments. These are then loaded within the context of HLA molecules belonging to class II of the major histocompatibility complex (MHC class II) expressed by dendritic cells that migrate to T-cell areas of regional thoracic lymph nodes where antigen presentation to T lymphocytes takes place. Recognition of specific antigenic peptides by T-cell receptors triggers sensitization and the following adaptive immune response. Allergen-dependent activation of naïve T lymphocytes requires the interaction of their costimulatory molecules (CD28, ICOS, and OX40) with the respective counter-ligands expressed by dendritic cells (CD80/B7.1, CD86/B7.2, ICOS ligand, and OX40 ligand) [20]. The type of antigen presentation-dependent polarization of T lymphocytes is critically determined by the cytokine milieu. In particular, Th2 differentiation needs a microenvironment characterized by high concentrations of IL-4 and low levels of IL-12. IL-4 is produced by mast cells and basophils but not by dendritic cells [21]. GATA3 is the key transcription factor, expressed by Th2 lymphocytes, that drives the synthesis of Th2 cytokines. The latter include IL-4, IL-5, IL-9, and IL-13. These cytokines stimulate the maturation and recruitment of other immune cells involved in the allergic cascade, such as eosinophils and mast cells [22]. In particular, IL-4 and IL-13 act on B lymphocytes by driving immunoglobulin class switching towards the production of IgE (Figure 1) [6, 8]. IL-9, secreted by a further subset of T lymphocytes (Th9) derived from Th2 cells, attracts mast cells and triggers their differentiation (Figure 1) [23].

IL-5 plays a pivotal role in inducing the differentiation and maturation of eosinophils in bone marrow, as well as their activation and survival [24]. Transendothelial migration of eosinophils to the airways of patients with allergic asthma is stimulated by IL-4 via induction of vascular cell adhesion molecule-1 (VCAM-1), which specifically interacts with its eosinophil counterligand very late antigen-4 (VLA-4). In chronic allergic asthma, IgE-activated mast cells persistently secrete eosinophil recruiting cytokines (e.g., IL-3, IL-4, IL-5, and GM-CSF), whose action is synergized by several chemokines such as eotaxins 1/2 (CCL11/CCL24) and RANTES (regulated upon activation, normal T cell expressed and secreted) [25, 26]. As a consequence, the prolonged eosinophil infiltration and degranulation result in a continuous release of cytotoxic products including major basic protein, eosinophil cationic protein, eosinophil-derived neurotoxin, and eosinophil peroxidase, which significantly contribute to airway epithelial damage, mucus overproduction from goblet cells, bronchial hyperresponsiveness, and impaired ciliary beating. Moreover, eosinophils secrete cysteinyl leukotrienes (LTC_4_, LTD_4_, and LTE_4_), powerful proinflammatory mediators that induce bronchoconstriction, increased vascular permeability, mucus hypersecretion, and activation of eosinophils themselves [27–30]. Furthermore, eosinophils synthesize growth factors such as transforming growth factor-*β* (TGF-*β*), thus participating in the pathogenesis of airway structural changes occurring in asthma such as thickening of epithelial basement membrane and smooth muscle hypertrophy [31]. In addition to producing many proinflammatory mediators, eosinophils contribute to allergic asthmatic inflammation also by acting as antigen presenting cells [32], thereby exposing allergen peptides to T lymphocytes.

The late-onset variant of eosinophilic asthma that occurs at adulthood is often nonallergic. Indeed, this particular disease subphenotype frequently develops in the absence of allergen-dependent activation of Th2 lymphocytes. Current evidence suggests that a central function in coordinating eosinophilic nonallergic asthma is exerted by type 2 innate lymphoid cells (ILC2s), whose differentiation depends on the expression of the transcription factor ROR*α* [9, 10, 12]. Following stimulation elicited by TSLP, IL-25, and IL-33, ILC2s release Th2-type cytokines, including large quantities of IL-5 and IL-13, but much less IL-4 [33, 34]. Production of Th2-type cytokines by these cells is also stimulated by prostaglandin D_2_ (PGD_2_) via activation of its CRTH2 (chemoattractant receptor-homologous molecule expressed on Th2 cells) receptor, which is expressed by ILC2s [35]. High levels of ILC2s have been found in eosinophilic nasal polyps, as well as in lung and peripheral blood of asthmatic patients [36, 37]. Furthermore, in murine experimental models of asthma induced by either influenza A virus or the fungus* Alternaria alternata*, characterized by airway eosinophilia, it was shown that ILC2s were required and sufficient to cause eosinophil recruitment and bronchial hyperresponsiveness, independently of Th2 cells [38, 39].

Therefore, two different and nonmutually exclusive proinflammatory pathways activated by either allergen-specific Th2 lymphocytes or allergen-independent innate ILC2s, both leading to IL-5 production, are responsible for eosinophilic airway inflammation in asthma. IL-5, together with other survival factors such as IL-3, GM-CSF, IL-25, IL-33, and TSLP, delays eosinophil apoptosis [26]. Eosinophil apoptosis is induced by lipoxin A4, an arachidonic acid metabolite that inhibits IL-5 production and whose levels have been found to be decreased in the exhaled breath condensate of patients with asthma undergoing disease exacerbations [26, 40]. From a therapeutic point of view, corticosteroids are powerful inducers of eosinophil apoptosis [41], and this proapoptotic action represents one of the most effective antiasthma mechanisms of these drugs. Inhaled corticosteroids, which are the cornerstone of asthma treatment, cause eosinophil apoptosis by suppressing the synthesis of important eosinophil survival factors such as IL-3, IL-5, and GM-CSF [42, 43]. However, some patients with severe, uncontrolled eosinophilic asthma can exhibit various degrees of corticosteroid resistance. In these cases, an alternative antieosinophil pharmacological approach might be based on the experimental use of biological drugs directed against IL-5 or its receptor [44–46]. Mepolizumab, a humanized anti-IL-5 monoclonal antibody, can effectively reduce eosinophil numbers in airways and blood [47, 48]. These effects were paralleled by significant reductions in the rate of asthma exacerbations, experienced by both allergic and nonallergic asthmatic patients [49, 50]. In addition to mepolizumab, another interesting anti-IL-5 biologic drug is reslizumab, an IgG4/*κ* humanized monoclonal antibody. When compared to placebo in patients with poorly controlled eosinophilic asthma, reslizumab has been recently shown to significantly decrease sputum eosinophils and improve lung function, as well as to induce a positive trend towards better asthma control [51]. The antiasthma effects of reslizumab were most pronounced in a subgroup of patients characterized by the highest levels of blood and sputum eosinophils, which were associated with the presence of nasal polyposis [51]. Benralizumab is an IgG1 monoclonal antibody directed to the *α* chain of the IL-5 receptor that can effectively decrease peripheral blood eosinophils and also reduce asthma exacerbations [52, 53]. Altogether, these observations clearly indicate that eosinophils are strategic cellular targets of paramount importance for the treatment of airway inflammation in both allergic and nonallergic asthma.

## 3. Neutrophilic Asthma

Whilst Th2 lymphocytes are mainly involved in the development of eosinophilic allergic asthma, other Th cell subsets induce airway neutrophilic inflammation, often associated with the most severe asthmatic phenotypes [54]. In particular, a specific lineage of CD4^+^ effector T lymphocytes, expressing IL-17 and thus named Th17, appears to play a key role in airway neutrophilia (Figure 1) [13, 55]. Indeed, in lung tissue sections from asthmatic patients there is an overexpression of IL-17A and IL-17F, whose levels correlate with asthma severity, especially in subjects with neutrophilic, steroid-resistant disease [56]. Differentiation of Th17 lymphocytes requires a composite mixture of cytokines and costimulatory signals, operating in the presence of high antigen concentrations that induce CD40 ligand (CD40L) expression on naïve T cells [57]. The interaction of CD40L with CD40 molecules exposed by dendritic cells also expressing CD86 will lead to Th17 polarization only within a cytokine milieu including IL-1*β*, IL-6, and TGF-*β* [15, 58]. These cytokines are responsible for T cell upregulation of the master transcription factor ROR*γ*t, which is specific for Th17 commitment, as well as the receptor for IL-23 [58, 59]. This latter cytokine is crucial for maintaining Th17 cells in a functionally active state [60]. Allergens and other environmental stimuli such as cigarette smoke and diesel exhaust particles have been shown to trigger Th17-mediated airway inflammation in asthmatic subjects [15]. Indeed, cigarette smoking is often associated with bronchial neutrophilia, more severe asthma, and corticosteroid insensitivity [61, 62]. In addition to cigarette smoke and airborne pollutants, other environmental agents such as microbes and microbial particles have also been implicated in the development of severe asthma [15]. In this regard, a relevant pathogenic role in Th17 cell-associated severe asthma could be played by the NLRP3 (nucleotide-binding oligomerization domain-like receptor family, pyrin domain containing 3 activation) inflammasome, an intracellular multiprotein complex that facilitates the autoactivation of the proinflammatory cysteine protease caspase-1 [63, 64]. NRLP3 is activated by serum amyloid A (SAA) protein, which is produced upon exposure of airway epithelial cells to microbes and is detectable at high concentrations in both serum and induced sputum of asthmatic patients [65]. Asthmatic subjects are very susceptible to airway microbial burden, and both viral and bacterial components act as pathogen-associated molecular patterns (PAMPs), which are recognized by Toll-like receptors (TLRs) [63, 64]. The latter activate the transcription factor NF-*κ*B (nuclear factor-*κ*B), which induces the expression of pro-IL-1*β* and pro-IL-18 cytokines. The subsequent assembly of the NLRP3 inflammasome leads to activation of caspase-1, which cleaves pro-IL-1*β* and pro-IL-18, thus converting them in their mature forms [63, 64]; through this mechanism, active IL-1*β* can thus contribute to promote Th17 cell-dependent inflammation. Such a cascade of intracellular events, leading to NLRP3 activation, can also be triggered by danger-associated molecular patterns (DAMPs) [66], alarm signals that, for example, originate as a consequence of airway epithelial damage induced by oxidative stress associated with cigarette smoking, and airborne pollutants. NLRP3 and caspase-1 protein have been found to be expressed in sputum neutrophils and macrophages from subjects with neutrophilic asthma [67]. Activation of the NLRP3 inflammasome can also occur in obesity-associated airway hyperresponsiveness [68].

Besides Th17 lymphocytes, other cellular sources of IL-17 include *γδ* T cells, cytotoxic T cells, invariant NK T cells, NK cells, and type 3 innate lymphoid cells (ILC3s) [12, 69]; the latter, which require ROR*γ*t and GATA3 transcription factors for their development, can be detected in the bronchoalveolar lavage fluid from patients with severe asthma [12, 70]. Whatever their cellular sources (e.g., Th17 cells and ILC3s) are, once released IL-17A and/or IL-17F stimulate airway structural cells, including bronchial epithelial cells and subepithelial fibroblasts to secrete powerful neutrophil chemoattractants such as IL-8 (CXCL8) and CXCL1/GRO-*α* [71–73]. These proinflammatory effects are mediated by stimulation of a receptor complex consisting of IL-17 receptor A (IL-17RA) and IL-17 receptor C (IL-17RC) subunits, coupled to a signaling network leading to NF-*κ*B activation [74]. Th17 cell-associated neutrophilic asthma is often characterized by severe clinical forms, which are very difficult to manage because of frequent steroid resistance. Indeed, differently from their proapoptotic action exerted on eosinophils, corticosteroids inhibit neutrophil apoptosis, thereby prolonging the survival of these inflammatory cells [75]. Thus, novel antineutrophil therapies are extremely needed for treatment of severe neutrophilic asthma. In this regard, a placebo-controlled trial has been recently carried out to evaluate the effects on moderate-to-severe uncontrolled asthma of brodalumab, a human IgG2 anti-IL-17RA monoclonal antibody that blocks the biological activity of both IL-17A and IL-17F [76]. According to this study, some clinical benefits were detected only in a subgroup of asthmatic patients exhibiting a high degree of airflow limitation reversibility in response to bronchodilator inhalation. These effects might also be promoted by brodalumab-induced neutralization of the biological actions of IL-25 (IL-17E), which are dependent on activation of the IL-17 receptor A/receptor B complex [74]. However, the reported decrease in asthmatic symptoms was not paralleled by a significant improvement in respiratory function [76]. Moreover, a relative increase in airway respiratory infections was also found [76] consistent with the well-known protective role of IL-17 in host defense against bacterial, viral, and fungal pathogens [69]. Therefore, further studies are needed to better evaluate the therapeutic potential of anti-IL-17 strategies in the management of difficult-to-treat, neutrophilic severe asthma.

Before the discovery of Th17 lymphocytes, Th1 cells, whose differentiation is driven by dendritic cells producing IL-12 and type 1 interferons and requires the expression of the specific transcription factor T-bet, were believed to be the main cellular coordinators of neutrophilic asthma [15, 77]. Indeed, Th1 cells and the Th1-derived cytokines interferon-*γ* (IFN-*γ*) and tumour necrosis factor-*α* (TNF-*α*) are increased in patients with severe asthma, and these observations suggested that Th1 cells could mediate airway neutrophilic inflammation (Figure 1) [15, 78]. However, a large study carried out in patients with persistent severe asthma receiving golimumab, a human anti-TNF-*α* monoclonal antibody, did not evidence any significant improvement in lung function and disease exacerbations [79]. Moreover, serious adverse infectious and neoplastic events like active tuberculosis, pneumonia, sepsis, and several different malignancies (breast cancer, B-cell lymphoma, metastatic melanoma, cervical carcinoma, renal cell carcinoma, basal cell carcinoma, and colon cancer) were reported [79]. Therefore, the trial was interrupted and it appears to be currently very unlikely that anti-TNF-*α* antibodies will be soon further evaluated for treatment of severe asthma.

## 4. Concluding Remarks

Airway inflammation, a key feature of asthma, is driven by complex cellular and molecular mechanisms leading to eosinophilic and/or neutrophilic bronchial infiltration, mainly dependent on Th2 and Th17 cell differentiation and activation, respectively (Figure 1). Within this pathophysiologic context a central role is played by dendritic cells, which direct the commitment of different Th cell lineages responsible for activation and recruitment into the airways of eosinophils and neutrophils [15]. Globally, these events occur in asthma as a consequence of the defective function of specific regulatory T lymphocytes (Treg cells) [80–83] (Figure 1). Indeed, because Treg cells directly or indirectly inhibit proinflammatory dendritic cells and promote the generation of tolerogenic dendritic cells, thus preventing the activation of effector Th1, Th2, and Th17 lymphocytes [82], the functional impairment of Treg-mediated immunomodulation makes asthmatic patients susceptible to the development of eosinophilic and neutrophilic bronchial inflammation. In particular, combined patterns of both neutrophilic and eosinophilic airway infiltrates may often coexist in severe asthma and in recurrent acute disease relapses that characterize the so-called exacerbation-prone asthmatic phenotype [84]. These exacerbations can be caused by allergens and especially by respiratory viruses, whose pathogenic effects within the airways of asthmatic subjects are favoured by a deficient epithelial synthesis of antiviral cytokines such as interferons-*β* (IFN-*β*) and -*λ* (IFN-*λ*) [85, 86].

In conclusion, it is undoubtable that better knowledge of the intricate pathobiologic networks underlying the onset and progression of airway inflammation in asthma will pave the way for substantial improvements in the treatment of this widespread and sometimes severe disease. In particular, future research should focus on the development of prevention strategies and therapeutic options aimed to restore the impaired balance between immunosuppressive Treg lymphocytes and the various branches of asthma-inducing adaptive immunity, especially referring to Th2- and Th17-mediated responses. Such efforts should widen and improve the currently available immunological and pharmacological approaches. In this regard, biologic therapies could eventually provide the tools for personalized antiasthma medications capable of satisfying the individual needs of patients expressing distinct disease phenotypes.

## Abbreviations

CXCL1: Chemokine CXC motif ligand 1
CXCL8: Chemokine CXC motif ligand 8
MMP: Matrix metalloproteinases
NE: Neutrophil endopeptidase
ROS: Reactive oxygen species
TNF-*α*: Tumour necrosis factor-*α*
TSLP: Thymic stromal lymphopoietin.
